# Quantifying in vitro *B. anthracis* growth and PA production and decay: a mathematical modelling approach

**DOI:** 10.1038/s41540-024-00357-1

**Published:** 2024-03-29

**Authors:** Bevelynn Williams, Jamie Paterson, Helena J. Rawsthorne-Manning, Polly-Anne Jeffrey, Joseph J. Gillard, Grant Lythe, Thomas R. Laws, Martín López-García

**Affiliations:** 1 Department of Applied Mathematics, School of Mathematics, University of Leeds, Leeds, UK; 2 CBR Division, Defence Science and Technology Laboratory, Salisbury, UK

**Keywords:** Computational biology and bioinformatics, Applied mathematics, Diseases, Immunology, Microbiology

## Abstract

Protective antigen (PA) is a protein produced by *Bacillus anthracis*. It forms part of the anthrax toxin and is a key immunogen in US and UK anthrax vaccines. In this study, we have conducted experiments to quantify PA in the supernatants of cultures of *B. anthracis* Sterne strain, which is the strain used in the manufacture of the UK anthrax vaccine. Then, for the first time, we quantify PA production and degradation via mathematical modelling and Bayesian statistical techniques, making use of this new experimental data as well as two other independent published data sets. We propose a single mathematical model, in terms of delay differential equations (DDEs), which can explain the in vitro dynamics of all three data sets. Since we did not heat activate the *B. anthracis* spores prior to inoculation, germination occurred much slower in our experiments, allowing us to calibrate two additional parameters with respect to the other data sets. Our model is able to distinguish between natural PA decay and that triggered by bacteria via proteases. There is promising consistency between the different independent data sets for most of the parameter estimates. The quantitative characterisation of *B. anthracis* PA production and degradation obtained here will contribute towards the ambition to include a realistic description of toxin dynamics, the host immune response, and anti-toxin treatments in future mechanistic models of anthrax infection.

## Introduction

*Bacillus anthracis* is the bacterial pathogen that causes anthrax. One of the characteristic virulence factors that contribute to the pathogenic success of *B. anthracis* is the production of three proteins that are collectively termed anthrax toxin: protective antigen (PA), oedema factor (EF), and lethal factor (LF). PA molecules bind to receptors on host cells and are cleaved in order to create a binding site for one of the active components, EF or LF. Although EF and LF are the components that exert a toxic effect on cells, they are not able to enter cells and cause this toxicity in the absence of PA molecules^1^. EF, in combination with PA forms the oedema toxin, and LF, in combination with PA forms the lethal toxin. These two toxins cause different cellular responses to suppress the host’s immune response during infection. Lethal toxin disrupts cell signalling pathways of macrophages and some other cells, leading to cell death. On the other hand, oedema toxin inhibits the phagocytosis of bacteria by neutrophils^2^. The toxin proteins are also critical components in two effective anthrax vaccines for humans: anthrax vaccine adsorbed (AVA) in the USA and anthrax vaccine precipitated (AVP) in the UK. These vaccines are composed of culture filtrates containing toxin proteins expressed by avirulent vaccine strains of *B. anthracis*. They aim to counter toxin action by initiating the body to generate antibodies against PA^3^.

Mechanistic mathematical models of infection can provide significant improvement in the understanding and quantification of key infection mechanisms. Novel multi-scale models recently developed for the bacterial pathogen *Francisella tularensis* have been parameterised using various experimental data sets, enabling the estimation of parameters that describe important aspects of its pathogenicity^4,5^. These models also allow predictions for the probability of response, and mean time until response, of an infected individual as a function of the initial infection dose. Data for *B. anthracis* infection is fairly scarce. In particular, an important ingredient is missing for the development of a within-host model that incorporates a quantitative description of the role played by the anthrax toxins during infection. The role of toxins was included in the model by Day et al.^6^ but the toxin level was only modelled in a qualitative way, without units. In this paper, we make use of three independent in vitro experimental data sets to obtain a quantitative mathematical description of PA production by *B. anthracis*. This will be instrumental for the future development of anthrax models that incorporate a fully mechanistic description of within-host toxin dynamics. The mathematical model proposed here focuses only on the PA component of anthrax toxin (and not EF or LF), since PA is the essential toxin component that facilitates binding of EF and LF to cell surfaces. Furthermore, PA is the component targeted by two FDA-approved specific anti-PA treatments (Raxibacumab and Obiltoxaximab). Thus, quantification of PA production and degradation is valuable for the development of mathematical models that incorporate this type of treatment.

Previous experimental studies have been conducted to determine the biological activities of the anthrax toxin proteins and evaluate the quantities expressed by different *B. anthracis* strains in culture conditions. For example, Zai et al.^7^ conducted experiments to explore the expression of PA and LF by the A16R strain and the Sterne strain of *B. anthracis*. Both these strains are un-encapsulated but retain the ability to produce the toxin proteins. The human anthrax vaccine in China uses live-attenuated *B. anthracis* spores of the A16R strain, whereas the Sterne strain is used for the manufacture of the UK acellular anthrax vaccine. In the experiments by Zai et al.^7^, the growth kinetics of the bacteria were observed to follow a sigmoidal growth curve, reaching a stationary phase of around 10^7^ viable cells per ml. They found that the amount of LF and PA increased as the bacteria grew, peaked after around 12–16 hours of bacterial growth, and then declined rapidly. This rapid decline may be caused by the downregulation of the toxin genes due to the depletion of glucose in the culture^8^. Zai et al.^7^ suggested that an accumulation of proteases in the culture could be an alternative reason for the decrease in the levels of toxin proteins. This is because it has been shown that certain proteases secreted by *B. anthracis*, such as immune inhibitor A1 (InhA1), can cleave the anthrax toxin proteins^9^. Indeed, when protease inhibitors were added to the culture, Zai et al.^7^ observed that the amount of PA and LF increased and then maintained a high level rather than decreasing. Charlton et al.^10^ have simulated the AVP vaccine manufacturing process, and measured the bacterial growth as well as PA and LF concentrations for up to 32 hours. They observed much higher levels of PA and LF in the culture supernatants compared with those of Zai et al.^7^, which could be due to different experimental methods. Charlton et al.^10^ also did not observe an increased breakdown of the toxin proteins after the peak. In their experiment, once the glucose was exhausted, the bacteria appeared to use amino acids as an alternative carbon source. In this study, we have conducted similar experiments to quantify PA in the supernatants of cultures of *B. anthracis* Sterne strain, which were carried out at the Defence Science and Technology Laboratory (Dstl, UK) in Porton Down. Our experimental methods differ in some ways with respect to those of Zai et al.^7^, and Charlton et al.^10^. A particular strength when using the experimental data to parameterise a mathematical model is that we did not heat activate the *B. anthracis* spores. This meant that germination occurred at a more natural rate, allowing us to calibrate additional model parameters that describe the germination dynamics of the spores.

We mathematically model the dynamics of PA production by *B. anthracis* in vitro via a system of delay differential equations (DDEs) and fit this model to data from two previously published in vitro experimental studies (Zai et al.^7^ and Charlton et al.^10^) and to the new experimental data set obtained at Dstl. The same mathematical model is used to explain the dynamics observed in each of the three data sets, just under slightly different assumptions to represent different experimental conditions. The model considers germination of *B. anthracis* spores, maturation of newly desporulated bacteria into vegetative bacteria, logistic growth of vegetative bacteria, production of PA by vegetative bacteria, and decay of PA (naturally and due to proteases produced by the bacteria). DDEs are considered to represent the delay of PA production and protease production. Furthermore, the model considers the depletion of nutrients (e.g. glucose) in the culture medium, where the production rate of PA is assumed to be proportional to these nutrient levels. The mathematical model is calibrated to each of the three data sets separately, using approximate Bayesian computation sequential Monte Carlo (ABC-SMC)^11^, and the parameter estimates obtained for the different data sets are compared. Our results show that most of the parameter estimates are fairly consistent across all experiments. However, in the Dstl experiment, the bacteria were able to divide faster and grow to a higher concentration, which is reflected in the corresponding parameter estimates. Also, the maximal per CFU production rate of PA is estimated to be higher for the Charlton et al.^10^ data set.

## Results

**Fig. 1:**
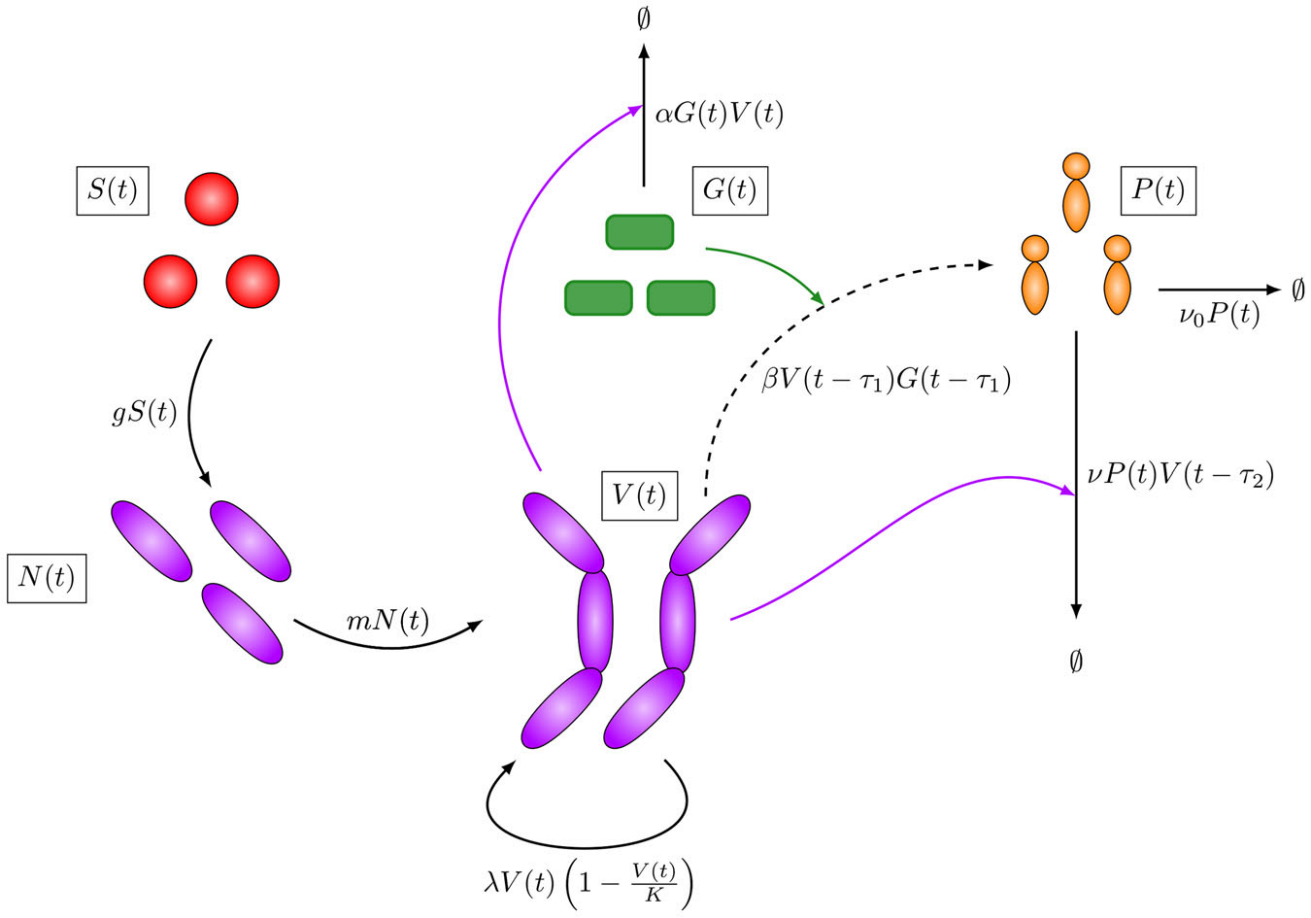
A schematic representation of the model. Black arrows represent species transitioning from one state to another, coloured arrows indicate that a population contributes to a particular reaction, and the dashed arrow represents PA production.

We fit the mathematical model in Fig. 1 (described in detail in the Methods section) to data from two previously published in vitro experimental studies (Zai et al.^7^ and Charlton et al.^10^) and to a new experimental data set obtained at Dstl. The experimental methods are described in the Methods section.

The parameter calibration is performed using the ABC-SMC algorithm^11^. The ABC method involves sampling parameter values from a chosen prior distribution, simulating the model using those parameter values, comparing the model output to the experimental data via a specified distance function, and accepting the parameter set into the posterior sample if the corresponding distance is less than some chosen threshold. ABC-SMC involves carrying out multiple iterations of the ABC algorithm, in order to refine posterior samples in each iteration while more efficiently exploring the parameter space. In particular, at each iteration, parameter values are sampled from the posterior distribution of the previous iteration and are perturbed with a kernel function. Here we use a component-wise uniform perturbation kernel, so that each component of the parameter set is perturbed independently in a uniform interval. The perturbed parameter set is then used to obtain a model prediction and is accepted if the distance between the model prediction and the data falls below the distance threshold for that iteration. We use a sequence of decreasing distance thresholds, such that the distance threshold at each iteration is some quantile of the distances from the accepted parameter sets in the previous iteration. In this manner, one obtains a set of distributions for the parameters that converge to the posterior distribution.

Uniform priors are used for each parameter (log-transformed in some cases) over the ranges in Table 1. During the model simulation step of the ABC-SMC algorithm, we add noise to each simulated data point, to take into account measurement errors in the observed data^12^. These added errors are independent Gaussian with zero mean and standard deviation equal to the standard deviation of the experimental data at the corresponding timepoint. Since the model is simultaneously being fitted to data sets of different types (bacterial CFU measurements and PA measurements) with different units (CFU vs ng/ml), it is necessary to ensure that the calculation of the distance between the model and the data is not affected by scale differences between the data types. Therefore, we define the following distances to compare model predictions with observed values.

1 $${D}_{1}=\mathop{\sum}\limits_{t}{\left({\log }_{10}\left(\frac{{B}_{t}^{* }}{B(t)}\right)\right)}^{2}+{\left({\log }_{10}\left(\frac{{S}_{t}^{* }}{S(t)+(1-f){S}_{0}^{* }}\right)\right)}^{2},$$

2 $${D}_{2}=\mathop{\sum}\limits_{t}{\left({P}_{t}^{* }-P(t)\right)}^{2},$$

where $S(t)+(1-f){S}_{0}^{* }$, *B*(*t*) = *N*(*t*) + *V*(*t*), and *P*(*t*) are the model predictions (see Eq. (6)) plus noise for the amount of spores (desporulating and dormant), total bacterial CFU, and PA, respectively, at time *t*. ${B}_{t}^{* }$ is the geometric mean observed number of bacterial CFU at time *t*, and ${P}_{t}^{* }$ is the mean amount of PA observed at time *t*. ${S}_{t}^{* }$ is the geometric mean observed number of spores at time *t*, which we only use for the Dstl data set. At each iteration of the ABC-SMC algorithm, two distance thresholds are generated, and parameter sets are only accepted if *D*_1_ and *D*_2_ both fall below their respective distance thresholds.

**Table 1:** Parameters in the mathematical model, along with their descriptions, units, and prior distributions

Parameter	Description	Unit	Prior
*f*	Fraction of initial spores that are able to germinate	-	*f* ~ *U*(0, 1)
*g*	Germination rate of spores into newly desporulated bacteria	h^−1^	${\log }_{10}g \sim U(-3,1)$
*ε*	Fraction of initial bacterial CFU that are newly desporulated	-	*ε* ~ *U*(0, 1)
*m*	Maturation rate of desporulated spores into vegetative bacteria	h^−1^	${\log }_{10}m \sim U(-3,1)$
*λ*	Rate of vegetative bacterial growth	h^−1^	${\log }_{10}\lambda \sim U(-1,1)$
*K*	Bacterial carrying capacity (per ml)	CFU	${\log }_{10}K \sim U(6,9)$
*α*	Rate that bacteria use up nutrients	(CFU·h)^−1^	${\log }_{10}\alpha \sim U(-12,-3)$
*β*	PA production rate	ng·(CFU·h)^−1^	${\log }_{10}\beta \sim U(-7,0)$
*ν*_0_	PA natural decay rate	h^−1^	${\log }_{10}{\nu }_{0} \sim U(-6,0)$
*ν*	PA decay rate due to proteases	(CFU·h)^−1^	${\log }_{10}\nu \sim U(-15,0)$
*τ*_1_	Time delay in PA production	h	*τ*_1_ ~ *U*(0, 15)
*τ*_2_	Time delay in protease production	h	*τ*_2_ ~ *U*(0, 24)

**Fig. 2:**
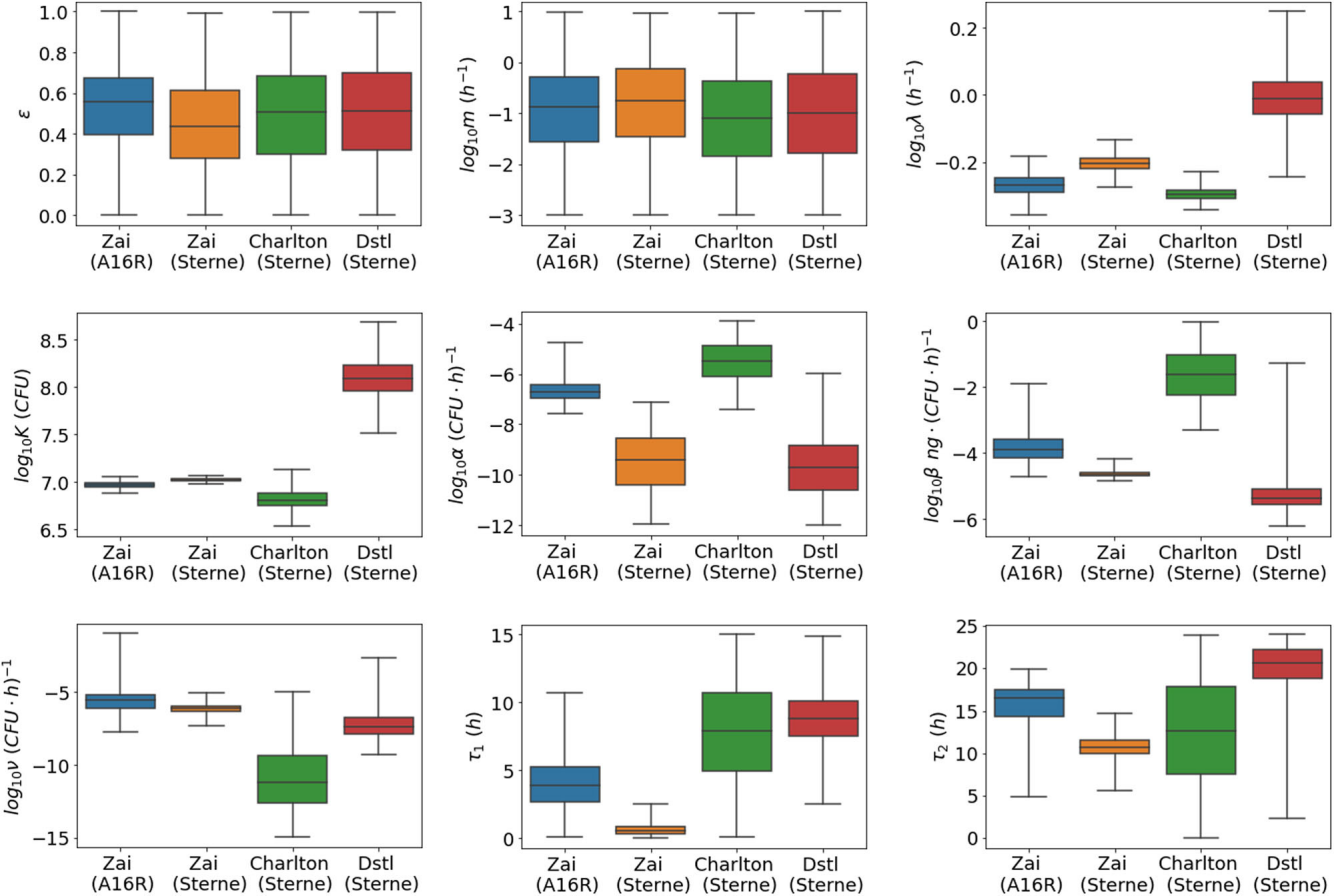
Box-plots comparing posterior distributions between data sets. The box-plots show the median, interquartile range, and range of each marginal posterior distribution.

In Table 2, we report summary statistics (median and 95% credible intervals) for the posterior distributions obtained from the different data sets, in order to facilitate comparisons. A visual comparison between the posterior distributions of each data set is shown in Fig. 2. For the parameters that determine the bacterial growth (the bacterial growth rate, *λ*, and the carrying capacity, *K*), the estimates from the Zai et al.^7^ and Charlton et al.^10^ data sets seem fairly consistent, while the estimates from the Dstl data seem to be slightly different. However, estimates for *λ* across all experimental data sets are roughly consistent with the growth rate estimate of *λ* = 0.89 per hour, which was obtained from the study by Kalns et al.^13^.

**Table 2:** A comparison between the medians and 95% credible intervals of the posterior distributions for each parameter after fitting the mathematical model to each data set

Parameter	Zai (A16R)	Zai (Sterne)	Charlton (Sterne)	Dstl (Sterne)
*f*	N/A	N/A	N/A	0.996 (0.988, 0.999)
*g*	N/A	N/A	N/A	1.92 (1.15, 4.79)
*ε*	0.55 (0.13, 0.87)	0.44 (0.05, 0.90)	0.51 (0.08, 0.91)	0.51 (0.06, 0.94)
*m*	0.14 (2 × 10^−3^, 4.6)	0.17 (3 × 10^−3^, 4.6)	0.08 (2 × 10^−3^, 4.7)	0.10 (2 × 10^−3^, 6.3)
*λ*	0.54 (0.47, 0.62)	0.63 (0.57, 0.68)	0.50 (0.47, 0.55)	0.97 (0.70, 1.34)
*K*	9 × 10^6^ (8 × 10^6^, 10^7^)	10^7^ (10^7^, 1.1 × 10^7^)	6 × 10^6^ (4 × 10^6^, 10^7^)	10^8^ (5 × 10^7^, 3 × 10^8^)
*α*	2 × 10^−7^ (5 × 10^−8^, 3 × 10^−6^)	4 × 10^−10^ (2 × 10^−12^, 3 × 10^−8^)	3 × 10^−6^ (10^−7^, 7 × 10^−5^)	2 × 10^−10^ (2 × 10^−12^, 7 × 10^−8^)
*β*	10^−4^ (3 × 10^−5^, 2 × 10^−3^)	2 × 10^−5^ (2 × 10^−5^, 4 × 10^−5^)	3 × 10^−2^ (10^−3^, 5 × 10^−1^)	4 × 10^−6^ (10^−6^, 2 × 10^−4^)
*ν*_0_	2 × 10^−4^ (3 × 10^−6^, 10^−2^)	**2** × **10**^−**4**^	**2** × **10**^−**4**^	**2** × **10**^−**4**^
*ν*	3 × 10^−6^ (4 × 10^−8^, 4 × 10^−5^)	7 × 10^−7^ (2 × 10^−7^, 3 × 10^−6^)	7 × 10^−12^ (3 × 10^−15^, 3 × 10^−7^)	4 × 10^−8^ (2 × 10^−9^, 5 × 10^−6^)
*τ*_1_	3.8 (0.8, 8.4)	0.5 (0.1, 1.7)	7.9 (1.3, 13.9)	8.8 (5.1, 13.5)
*τ*_2_	16.5 (7.9, 19.2)	10.8 (8.7, 13.5)	12.6 (1.6, 22.7)	20.6 (14.0, 23.8)

The values of *ν*_0_ in bold indicate fixed values that were used.

For parameters that influence the PA production and degradation (*α*, *β*, *ν*), estimates from the Zai et al.^7^ and Dstl data seem consistent, while estimates from the Charlton et al.^10^ data differ slightly. Estimates for the delay parameter *τ*_1_ are consistent between the Charlton et al.^10^ and Dstl data sets, whereas the delay until PA production is estimated to be much shorter for the Zai et al.^7^ data, possibly due to the fact that the bacteria had already been growing for 24 hours prior to inoculation. The delay until protease production, *τ*_2_, is estimated to be between 10 and 25 hours across all data sets.

When comparing the parameter estimates for the Sterne and A16R strains (experiments from Zai et al.^7^), we can observe that the Sterne strain bacteria seem to divide slightly faster (i.e., larger *λ*) and grow to a higher concentration (i.e., larger *K*), which is consistent with findings and discussions in ref. ^7^. On the other hand, we obtain rather different estimates for the rate, *α*, at which nutrients are consumed by the bacteria. This is because there are two different areas of parameter space that can capture the observed Sterne strain data, which accounts for the variability in the posterior distribution of *α*. These two areas roughly correspond to two different mechanisms which could explain the rapid decay in PA concentration observed in these data sets at late times. Differences between the predicted PA dynamics -and corresponding parameter estimates- for the two strains are discussed in detail in the following section.

Model fits to the data and kernel density estimates of the posterior distributions for each data set and parameter are shown in the following sections, where differences between data sets and possible reasons for these differences are discussed in more detail.

### Zai et al.^7^ data set

Zai et al.^7^ conducted experiments (described in the Methods section) using two different strains of *B. anthracis*—A16R and Sterne. For each strain separately, we fit the mathematical model (see Eq. (6)) to the data of viable counts and PA concentrations that they obtained, shown in ref. ^7^ [Figs. 1B and 4A, respectively].

For each strain, the first data measurements given are at 4 hours, and the initial conditions are not specified. We have assumed that there would initially be no PA present. In order to set the initial condition for the number of bacteria, we have fixed the number of bacteria at 4 hours in the model to be equal to the data point at that time. Then by assuming exponential growth of vegetative bacteria in the time interval 0–4 hours, we have worked backwards to obtain the initial condition

3 $${B}_{0}^{* }=\frac{{B}_{4}^{* }}{\left(1-\frac{\lambda \varepsilon }{\lambda +m}\right){e}^{4\lambda }+\frac{\lambda \varepsilon }{\lambda +m}{e}^{-4m}},$$

where ${B}_{4}^{* }$ is the data value at 4 hours. Furthermore, we have assumed that there are no desporulating spores present, since it is reasonable to assume that all desporulating spores would have already desporulated during the 24 hours of bacterial growth prior to inoculation into the assay culture. This means that we do not calibrate the parameters *f* and *g* for the Zai et al.^7^ data sets, since these parameters determine the dynamics of desporulating spores. The initial conditions of the model are taken to be, *S*(0) = 0, $N(0)=\varepsilon {B}_{0}^{* }$ (where ${B}_{0}^{* }$ is calculated from Eq. (3)), $V(0)=(1-\varepsilon ){B}_{0}^{* }$, *G*(0) = 1, and *P*(0) = 0.

#### A16R strain

For the A16R strain, an additional experiment was carried out by Zai et al.^7^, where a protease inhibitors cocktail was added to the LB culture medium. The measurements of PA concentration corresponding to this experiment are shown in ref. ^7^ [Fig. 7]. In order to model the PA concentration in this experiment, we add the following equation to the system in Eq. (6),

4 $$\frac{d{P}_{i}(t)}{dt}=\beta V(t-{\tau }_{1})G(t-{\tau }_{1})-{\nu }_{0}{P}_{i}(t),$$

with initial condition *P*_*i*_(0) = 0. The variable *P*_*i*_ represents the PA concentration in an experiment in which protease inhibitors have been added. This follows the same equation as *P*(*t*), but we set *ν* = 0 to represent the assumption that proteases will not be contributing to the degradation of PA in the experiment with protease inhibitors. To include this additional model variable and data into the distance function, we have

5 $${D}_{2}=\mathop{\sum}\limits_{t}{\left({P}_{t}^{* }-P(t)\right)}^{2}+{\left({P}_{i,t}^{* }-{P}_{i}(t)\right)}^{2},$$

where *P*_*i*_(*t*) is the model prediction (plus noise) for the amount of PA in an experiment with protease inhibitors at time *t* and ${P}_{i,t}^{* }$ is the mean amount of PA observed at time *t* in the experiment by Zai et al.^7^ using the A16R strain with protease inhibitors.

**Fig. 3:**
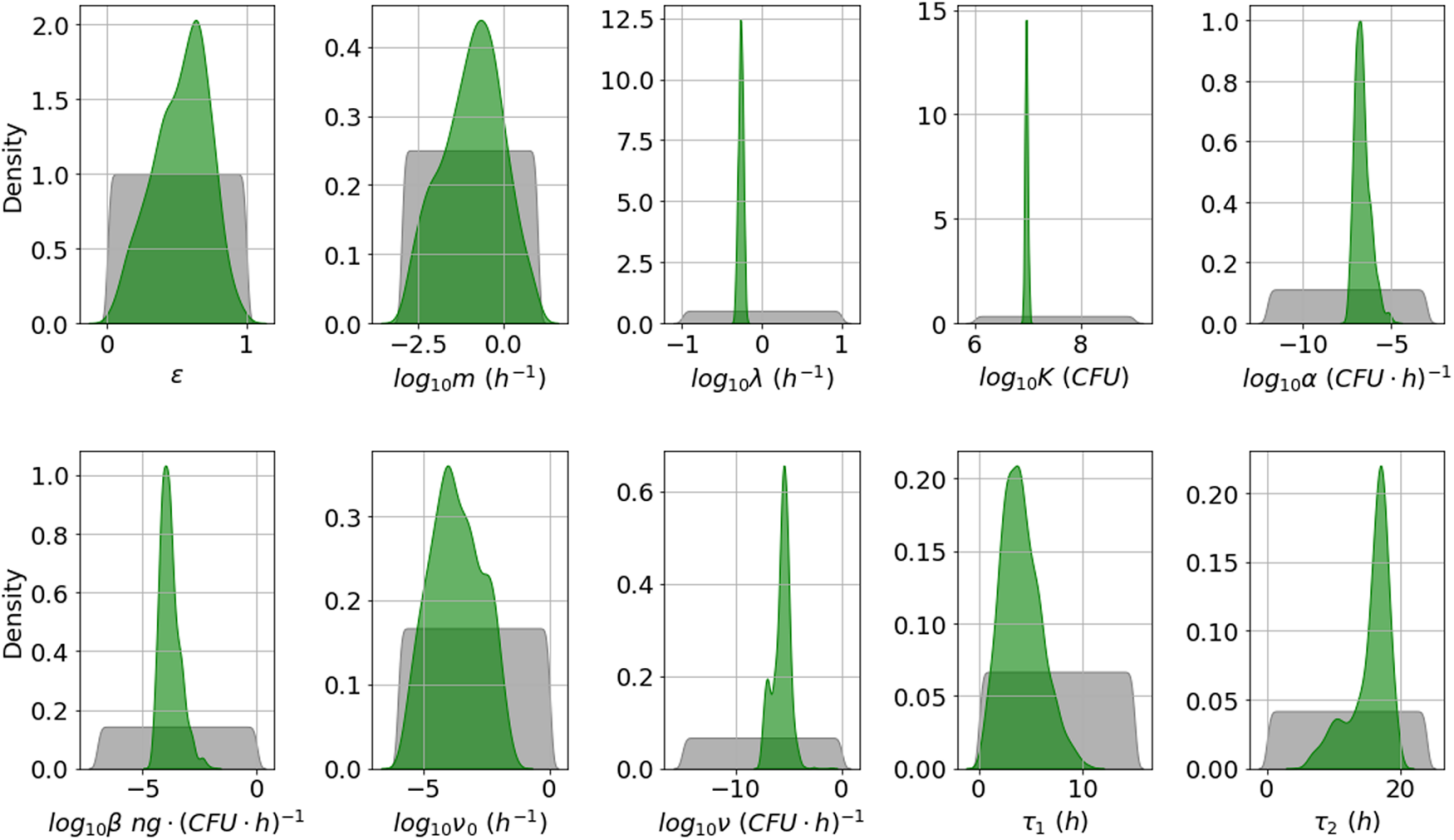
Posterior distribution corresponding to the Zai et al. A16R data set. Prior distributions are shown in grey and kernel density estimates of the marginal posterior distribution of each parameter in green. This posterior distribution was obtained by fitting the model in Eq. (6) to the A16R strain data from Zai et al.^7^.

From the marginal posterior distributions of each parameter, presented in Fig. 3, one can see that it has been possible to learn significantly about most of the parameters. However, *ε* and *m* have relatively wide posterior distributions. These parameters determine the fraction of bacterial CFU that are initially newly desporulated bacteria, and the rate at which these progress into vegetative bacteria, respectively. We have not been able to learn significantly about these parameters, since the type of data used here does not allow one to distinguish between newly germinated and vegetative bacterial CFU. Some parameter pairs are significantly correlated in the posterior sample. For example, *α* and *β* have a correlation coefficient approaching 1, and each has a correlation coefficient with *τ*_1_ of around 0.96. The parameter *α* determines how quickly nutrients are depleted in the media and therefore, how quickly the per CFU production rate of PA reduces over time, while the parameter *β* represents the initial (maximal) per CFU production rate of PA. The delay parameter *τ*_1_ is equivalent to the time, *t*, that the overall PA production rate changes from zero to *βV*(0). Therefore, a similar time-dependent PA concentration can be obtained by increasing or decreasing these three parameters simultaneously. Hence these parameters are not individually identifiable, given the data used.

**Fig. 4:**
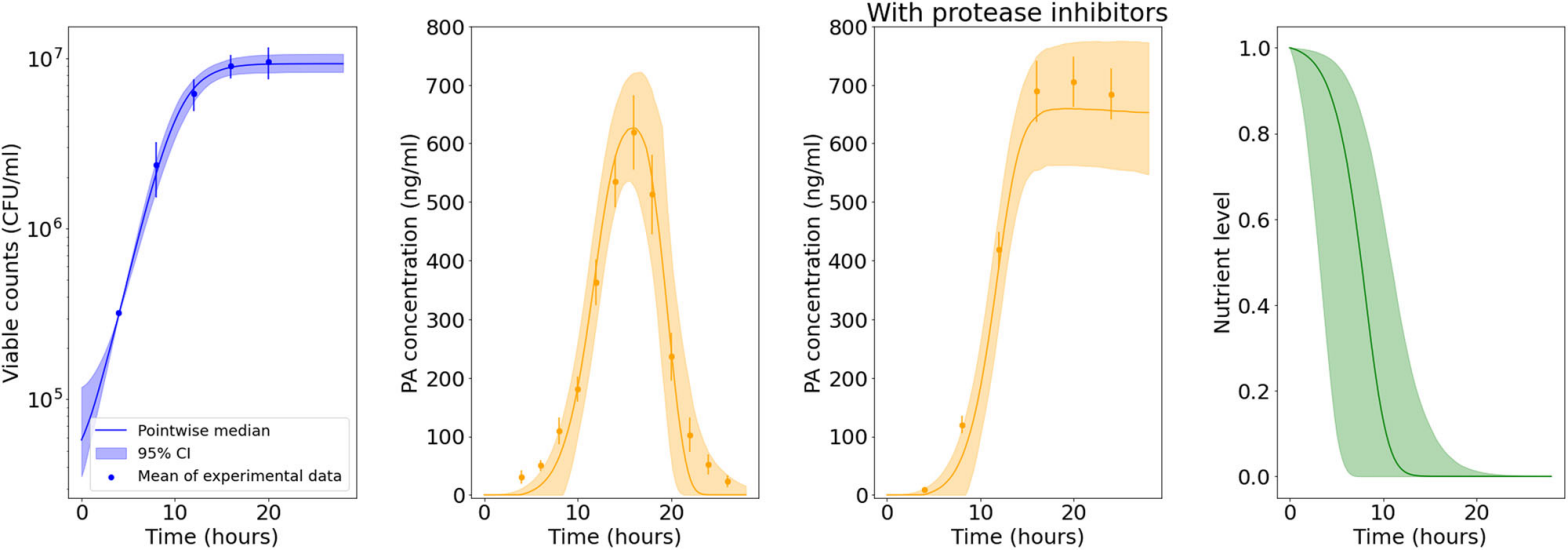
Model posterior predictions corresponding to the Zai et al. A16R data set. Pointwise medians (solid lines) and 95% credible intervals (shaded regions) of the model posterior predictions are shown for B(t) =  N(t) + V(t), P(t), P_i_(t), and G(t) (from left to right, respectively) using the parameter posterior distribution in Fig. 3. The A16R strain experimental data used to fit the model are presented as mean ± standard error (SEM) from three independent experiment runs, extracted from ref. ^7^ [Fig. 1B (viable counts), Fig. 4A (PA concentration), and Fig. 7 (PA concentration in the presence of protease inhibitors)].

Figure 4 shows the predicted amount of bacteria and PA versus the in vitro observations for the A16R strain. The solid lines represent the pointwise median of the model predictions from all parameter estimates in the posterior sample obtained via ABC-SMC, and the shaded regions represent the 95% credible intervals (CI) of these model predictions. Model predictions seem to agree well with data for all variables, where our mathematical model is able to successfully explain the exponential bacterial growth reaching a carrying capacity, the increase and peak of PA concentration, and the impact of protease inhibitors in preventing a rapid decline in PA concentration. Figure 4 also shows the posterior predictions for the nutrient level. The nutrients are consumed by the bacteria, and the depletion of nutrients reduces the PA production rate, resulting in the levelling off of the PA concentration in the predictions corresponding to the experiment with protease inhibitors.

#### Sterne strain

From the measurements in ref. ^7^ [Figs. 1B and 4A], the Sterne strain bacteria seem to replicate faster and produce more PA than the A16R strain. Hence we would expect most of the model parameter values to differ slightly between the two strains. The data from the experiment with protease inhibitors was very useful in the A16R calibration because the absence of protease effects on the PA decay allowed us to more accurately estimate the production rate of PA. This kind of data is not available for the Sterne strain. However, the value of the natural decay rate of PA, *ν*_0_, which was estimated using the A16R data, should be intrinsic to the PA protein itself and, in theory would not change depending on which strain produced the PA. Therefore, we leverage the information on the natural decay rate of PA obtained from the A16R calibration to set a value for this parameter when calibrating the model to the other data sets. In particular, from now on we set *ν*_0_ to be equal to the median value from the posterior in Fig. 3, giving *ν*_0_ = 2 × 10^−4^ *h*^−1^.

**Fig. 5:**
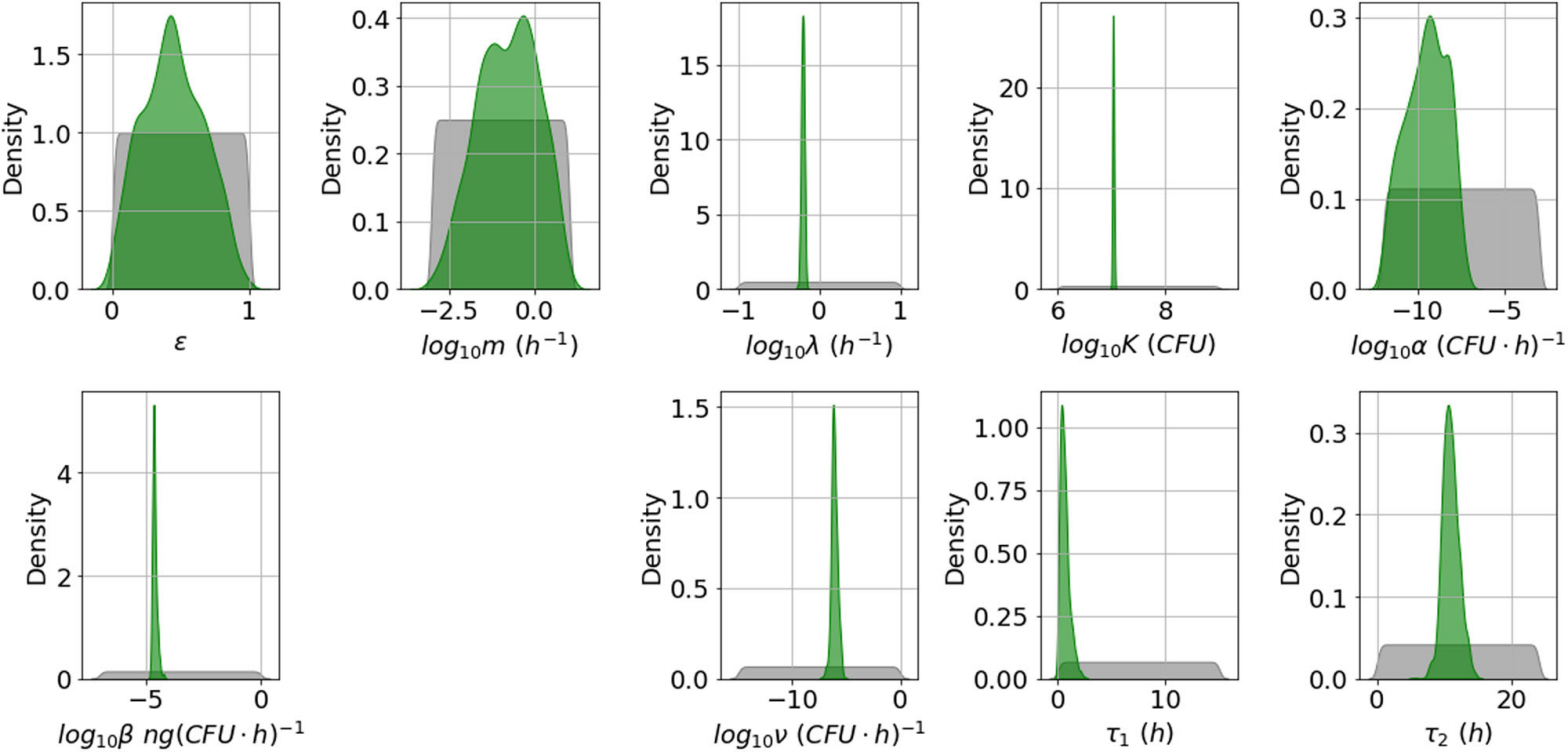
Posterior distribution corresponding to the Zai et al. Sterne data set. Prior distributions are shown in grey and kernel density estimates of the marginal posterior distribution of each parameter in green. This posterior distribution was obtained by fitting the model in Eq. (6) to the Sterne strain data from Zai et al.^7^.

**Fig. 6:**
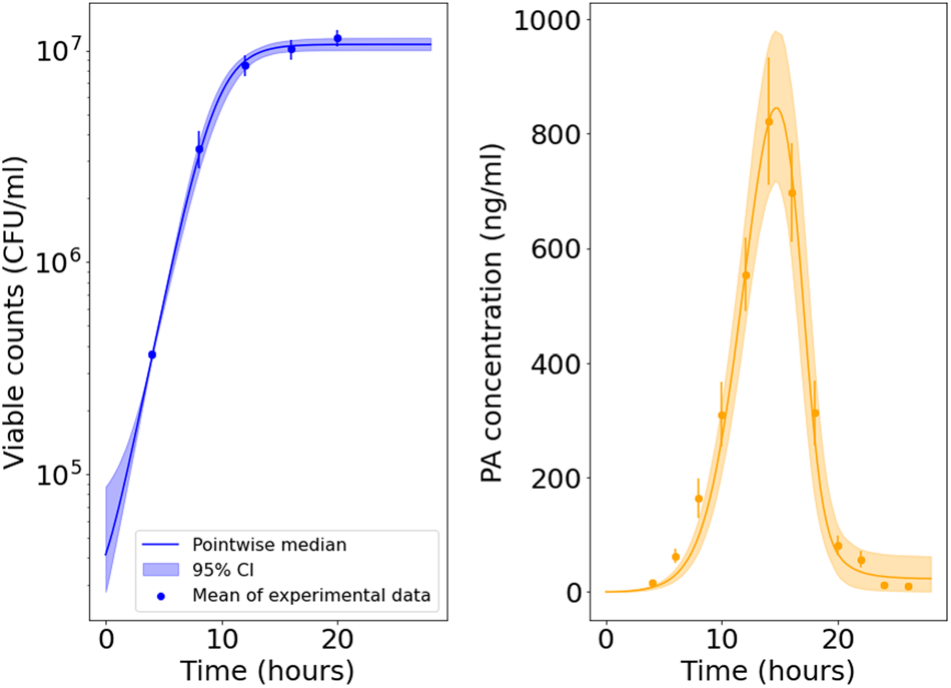
Model posterior predictions corresponding to the Zai et al. Sterne data set. Pointwise medians (solid lines) and 95% credible intervals (shaded regions) of the model posterior predictions are shown for B(t) = N(t) + V(t) (left) and P(t) (right), using the parameter posterior distribution in Fig. 5. The Sterne strain experimental data used to fit the model are presented as mean ± SEM from three independent experiment runs, extracted from ref. ^7^ [Fig. 1B (viable counts) and Fig. 4A (PA concentration)].

In general, the posterior distributions in Fig. 5 for the Sterne strain calibration are narrower than the ones in Fig. 3 for the A16R strain, and this is reflected in narrower 95% credible intervals for the posterior predictions in Fig. 6. It can be observed from the viable counts that the Sterne strain bacteria seem to divide slightly faster and grow to a higher concentration, which is reflected in the posterior distributions of the corresponding parameters, *λ* and *K*. Furthermore, the PA concentration data shows a higher peak for the Sterne strain than the A16R strain. Although the maximal per CFU PA production rate, *β*, is not estimated to be larger in the Sterne strain experiment, the rate of depletion of nutrients, *α*, as well as the delay until PA production is initiated, *τ*_1_, are estimated to be very small. This results in model predictions that show an increased PA yield. However, these parameter estimates lead to very different predictions for the nutrient level and for the PA concentration in the presence of protease inhibitors compared to those for the A16R strain in Fig. 4.

**Fig. 7:**
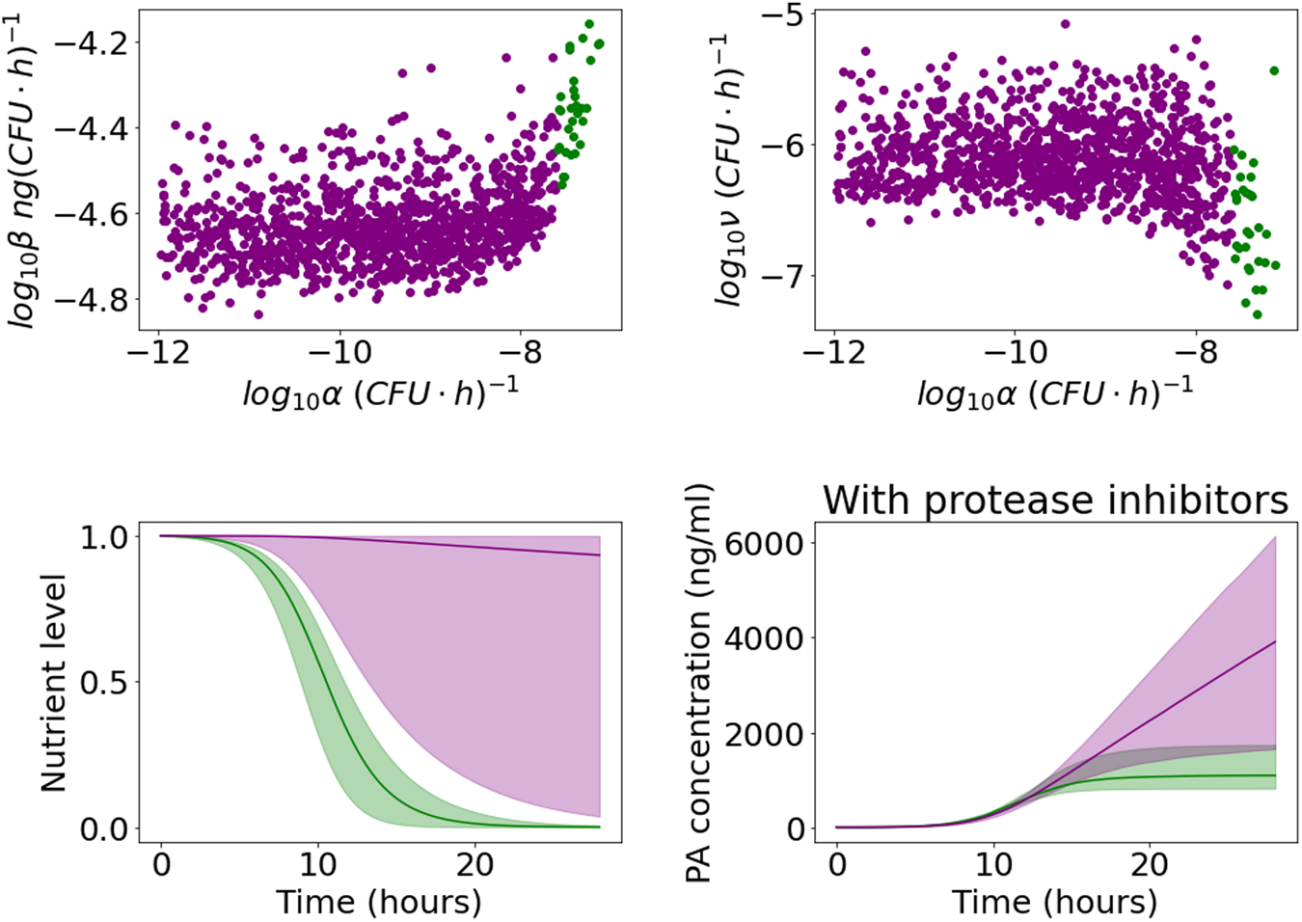
Distinguishing between parameter regimes corresponding to a fast or slow decline in nutrient level, for the Zai et al. Sterne posterior distribution. Top row: scatter plots of the parameter sets in the posterior sample from the calibration with the Zai et al.^7^ Sterne strain data set. The value of *α* is plotted on the *x* axis, and on the *y* axis is *β* (left) or *ν* (right). Points are coloured green if the value of *α* is larger than the minimum value of *α* in the posterior sample from the calibration with Zai et al.^7^ A16R strain data set (${\log }_{10}\alpha =-7.6$), and are coloured purple if the value of *α* falls below this threshold. Bottom row: pointwise medians (solid lines) and 95% credible intervals (shaded regions) of the model posterior predictions for *G*(*t*) (left) and *P*_*i*_(*t*) (right) using the posterior parameter sets with ${\log }_{10}\alpha \ge -7.6$ (green) or ${\log }_{10}\alpha < -7.6$ (purple).

In Fig. 7, we distinguish between two parameter regions in the posterior sample that both capture the observed data, but lead to rather different predictions for unobserved variables. In the scatter plots in Fig. 7, the points represent individual parameter sets in the Sterne strain posterior sample and are coloured according to whether the value of *α* is below the minimum accepted value in the A16R strain posterior sample. There is no significant correlation between the value of *α* and the value of *β* or *ν* in the region where *α* is small (purple), but when the value of *α* falls within the range accepted for the A16R strain (green), a positive correlation between *α* and *β*, and a negative correlation between *α* and *ν*, emerge. This is because if nutrients are consumed more quickly, a larger maximal PA production rate and a slower rate of PA degradation are needed to describe the data. In the bottom row of Fig. 7, one can see that for the parameter sets with large values of *α*, the nutrient level decreases to zero, similar to the predictions corresponding to the A16R strain data set in Fig. 4. In this case, PA production eventually stops, and the PA concentration subsequently declines due to PA degradation. On the other hand, when the nutrients are consumed very slowly, the impact of PA degradation by proteases is estimated to be much larger, in order for the model predictions to still capture the observed decline in PA concentration. If *ν* is set to zero to represent the presence of protease inhibitors, the first case (with larger values of *α*) predicts a levelling off of the PA concentration, similar to that observed in the experiment by Zai et al.^7^ using the A16R strain. For the parameter sets with smaller values of *α*, the model instead predicts a much higher PA concentration in the presence of protease inhibitors, which seems unlikely to be realistic, given the observations from the A16R strain experiment. Thus, although data from an experiment using protease inhibitors would be essential in order to determine which of these two cases better reflects reality, our results and parameter estimates seem more consistent across the different experimental data sets when the depletion of nutrients is the main cause behind the observed rapid decay in PA levels at late times (i.e., green parameter estimates in Fig. 7).

### Charlton et al.^10^ data set

Here we fit the mathematical model (see Eq. (6)) to the data of bacterial counts and PA concentrations obtained by Charlton et al., presented in ref. ^10^ [Figs. 1 and 4]. It can be seen in ref. ^10^ [Fig. 1] that the spore counts of each bottle remained fairly constant throughout the 32 hours, at ~30% of the number used to inoculate each bottle. This is likely to be because the spores were heat activated prior to inoculation, so they would have germinated quickly on contact with the glucose and amino acids of the culture media, and therefore the only remaining spores by the time the first CFU measurements were obtained were those spores that would not go on to germinate during the timescale of the experiment. Therefore the spore data are not used in the subsequent model calibration, and thus we do not calibrate *f* and *g* from this experiment.

Similarly to the Zai et al.^7^ data, we have fixed the number of bacteria at 2 hours in the model to be equal to the data point at that time. We then set the initial conditions to *S*(0) = 0, $N(0)=\varepsilon {B}_{0}^{* }$, $V(0)=(1-\varepsilon ){B}_{0}^{* }$, and *P*(0) = 0, where

$${B}_{0}^{* }=\frac{{B}_{2}^{* }}{\left(1-\frac{\lambda \varepsilon }{\lambda +m}\right){e}^{2\lambda }+\frac{\lambda \varepsilon }{\lambda +m}{e}^{-2m}}.$$

Once again, we fix the value of the natural PA decay rate to *ν*_0_ = 2 × 10^−4^ *h*^−1^, which is the median value obtained from the posterior in Fig. 3.

**Fig. 8:**
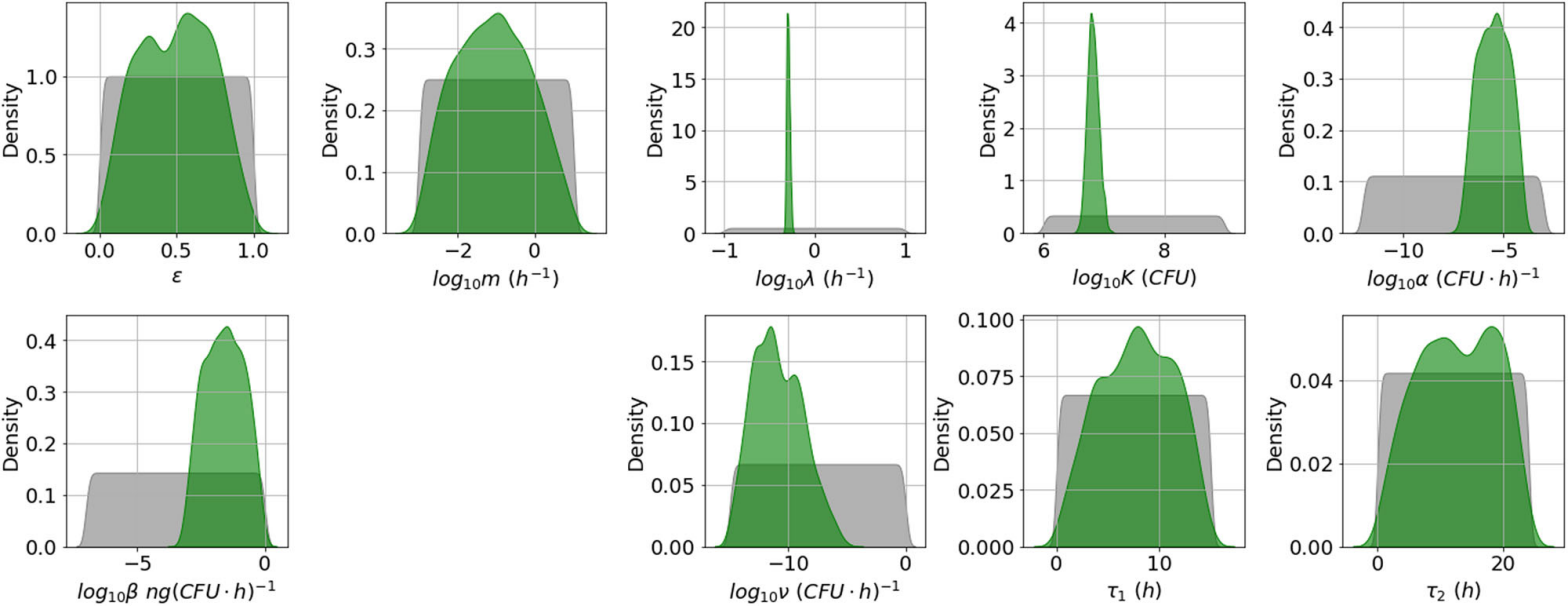
Posterior distribution corresponding to the Charlton et al. data set. Prior distributions are shown in grey and kernel density estimates of the marginal posterior distribution of each parameter in green. This posterior distribution was obtained by fitting the model in Eq. (6) to the Charlton et al.^10^ data.

By comparing the posterior distributions in Fig. 8 to those in Figs. 3 and 5, one can see that the estimates for most parameters are fairly consistent between the experiments of Charlton et al.^10^ and Zai et al.^7^. However, the rate of depletion of nutrients, *α*, and the maximal per CFU PA production rate, *β*, are estimated to be higher in the Charlton et al.^10^ experiment. It has been found that agitation can influence PA production by *B. anthracis*, possibly due to a change in the dissolved oxygen concentration of the assay culture^14^. Therefore, a possible explanation for the increase in PA production rate could be the method of static incubation implemented by Charlton et al.^10^.

**Fig. 9:**
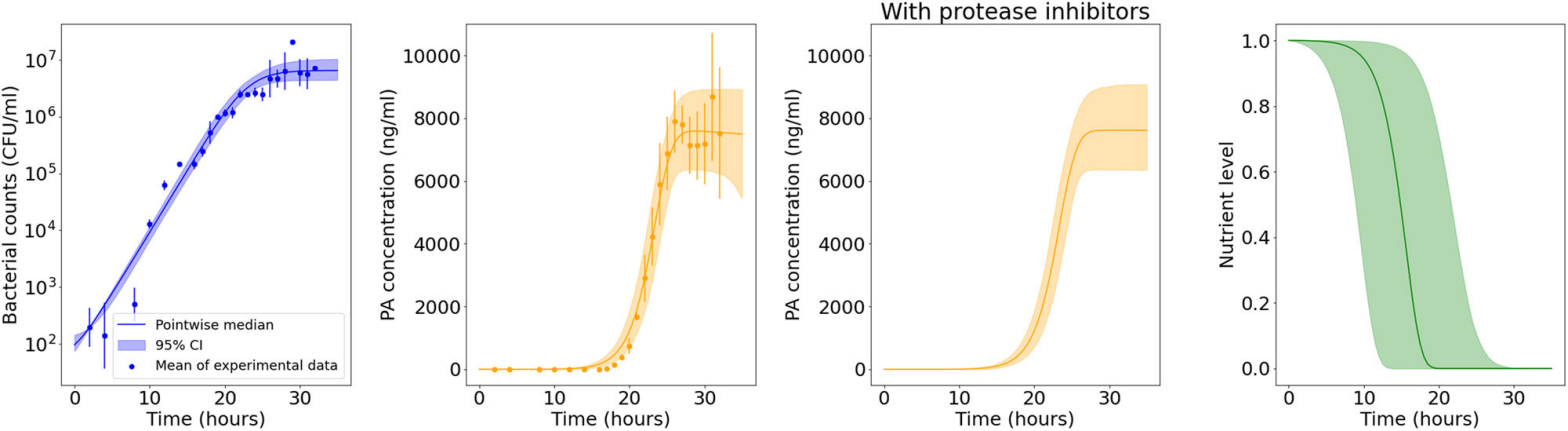
Model posterior predictions corresponding to the Charlton et al. data set. Pointwise medians (solid lines) and 95% credible intervals (shaded regions) of the model posterior predictions are shown for B(t) = N(t) + V(t), P(t), P_i_(t), and G(t) (from left to right, respectively) using the parameter posterior distribution in Fig. 8. The experimental data used to fit the model are presented as mean ± standard deviation from three independent Thompson bottles, obtained from ref. ^10^ [Fig. 1 (bacterial counts = viable counts−spore counts) and Fig. 4 (PA concentration)].

The parameter *ν* representing the rate of PA decay triggered by bacterial proteases is estimated to be lower in the Charlton et al.^10^ experiment than in the Zai et al.^7^ experiments, and has a relatively wide posterior distribution. This is because a rapid decay in PA concentration has not been observed in the Charlton et al.^10^ data set, which is crucial for the estimation of *ν*. Furthermore, due to the absence of an observed peak and subsequent decline in PA concentration, calibration with the Charlton et al.^10^ data set has not allowed us to learn about the value of *τ*_2_, representing the delay in the production of proteases by the bacteria. In Fig. 9, the model predictions of PA concentration corresponding to an experiment in which proteases are inhibited (i.e., *ν* = 0) are not significantly altered compared to the predictions with non-zero values of *ν*. This is because most parameter sets in the posterior sample have very large values of *τ*_2_ or very small values of *ν*, resulting in a low decay rate of PA. Hence, these results predict that the plateau in the PA concentration observed in the experiment is likely to be due to depletion of nutrients rather than PA degradation. However, the model predictions and data presented in Fig. 9 also show that the bacterial growth curve takes longer to reach the carrying capacity, compared with the Zai et al.^7^ experiments, since the initial amount of bacterial CFU is several orders of magnitude lower. Therefore, it could be that the accumulation of proteases is also delayed in this experiment, which has not been measured. Thus, it cannot be discounted that if measurements had been taken beyond 32*h*, a PA decay might have been observed.

### Dstl data set

Here we fit the mathematical model (see Eq. (6)) to the Dstl data set obtained in this study, which is reported in Table 3. In this study, the spores were not heat treated prior to inoculation into the assay culture and were observed to germinate much more slowly than in the Charlton et al.^10^ experiment. This has allowed us to make use of the spore counts to additionally calibrate the germination rate of spores, *g*, as well as the fraction of initial spores that are able to germinate, *f*. Furthermore, initial spore and bacterial counts were obtained as soon as possible after inoculation (1–2 minutes), so that we were able to fix the initial conditions of the model using experimental measurements, instead of inferring them by using a later time point. The initial conditions are therefore set to $S(0)=f{S}_{0}^{* },\,N(0)=\varepsilon {B}_{0}^{* },\,V(0)=(1-\varepsilon ){B}_{0}^{* },\,G(0)=1,\,P(0)=0,$ where ${S}_{0}^{* }$ and ${B}_{0}^{* }$ are the number of spores and bacterial CFU measured at time zero, respectively. Once again, we fix the value of the natural PA decay rate to *ν*_0_ = 2 × 10^−4^ *h*^−1^.

**Table 3:** Data for the spore counts, bacterial counts, and PA concentration (mean ± standard deviation) obtained at Dstl following the experimental methods in the Methods section

Time (hours)	log_10_(spores/ml)	log_10_(bacterial CFU/ml)	PA (ng/ml)
0	4.44 ± 0.09	4.34 ± 0.20	-
1.5	3.33 ± 0.14	4.73 ± 0.10	-
3.5	2.25 ± 0.18	5.48 ± 0.24	-
4.25	1.96 ± 0.59	5.74 ± 0.19	-
5	1.44 ± 0.12	6.20 ± 0.23	-
6	1.98 ± 0.64	7.05 ± 0.02	-
7	1.64 ± 0.66	7.45 ± 0.12	-
16	-	7.94 ± 0.06	194 ± 24
18	-	8.02 ± 0.03	386 ± 161
20	-	8.14 ± 0.02	1227 ± 511
22	-	8.18 ± 0.06	1950 ± 403
24	-	8.21 ± 0.14	2359 ± 289
40	-	7.96 ± 0.07	161 ± 0

**Fig. 10:**
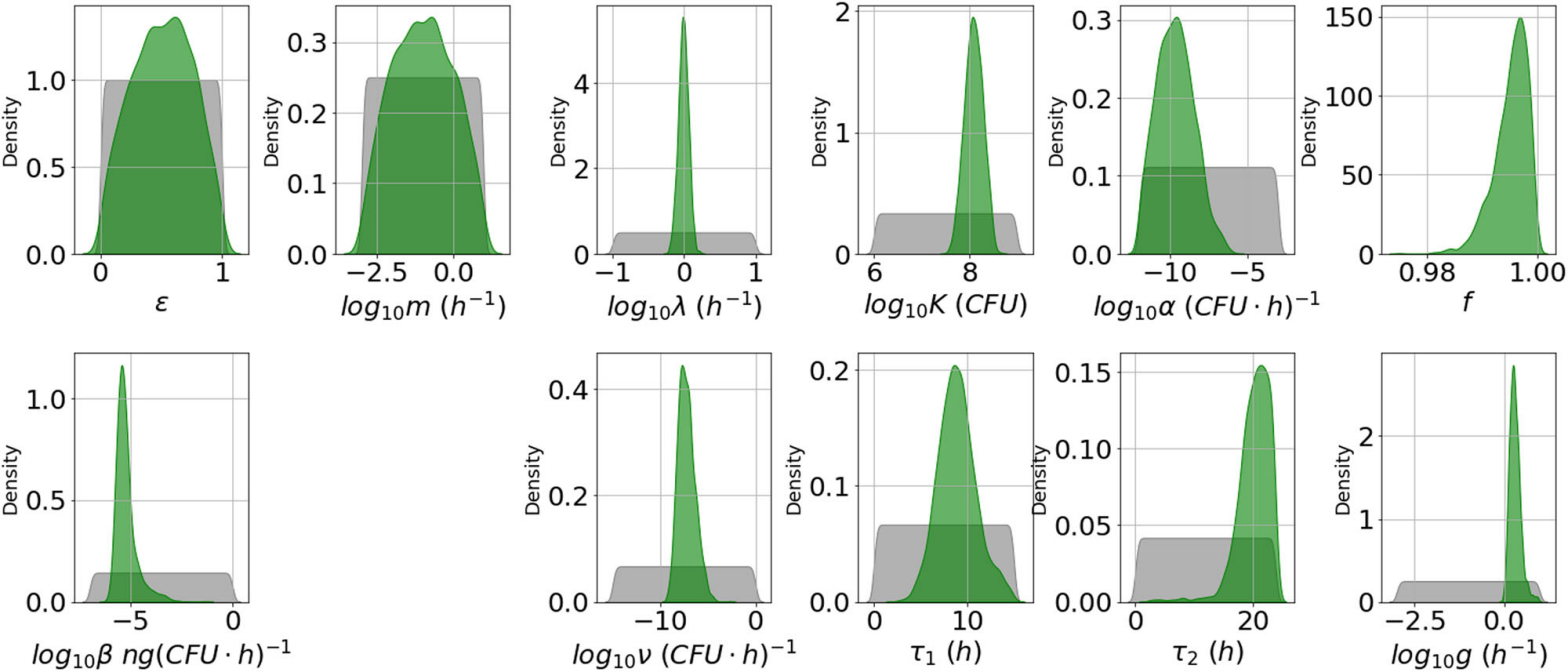
Posterior distribution corresponding to the Dstl data set. Prior distributions are shown in grey and kernel density estimates of the marginal posterior distribution of each parameter in green. This posterior distribution was obtained by fitting the model in Eq. (6) to the Dstl data in Table 3.

The marginal posterior distributions are shown in Fig. 10. Estimates for the germination rate, *g*, are slightly higher than previous estimates of this rate obtained by Williams et al.^15^, but the order of magnitude is similar. Furthermore, our results predict that almost all initial spores will germinate, with *f* estimated to be very close to 1. This is in contrast to the observations from Charlton et al.^10^, where ~30% of spores did not germinate.

**Fig. 11:**
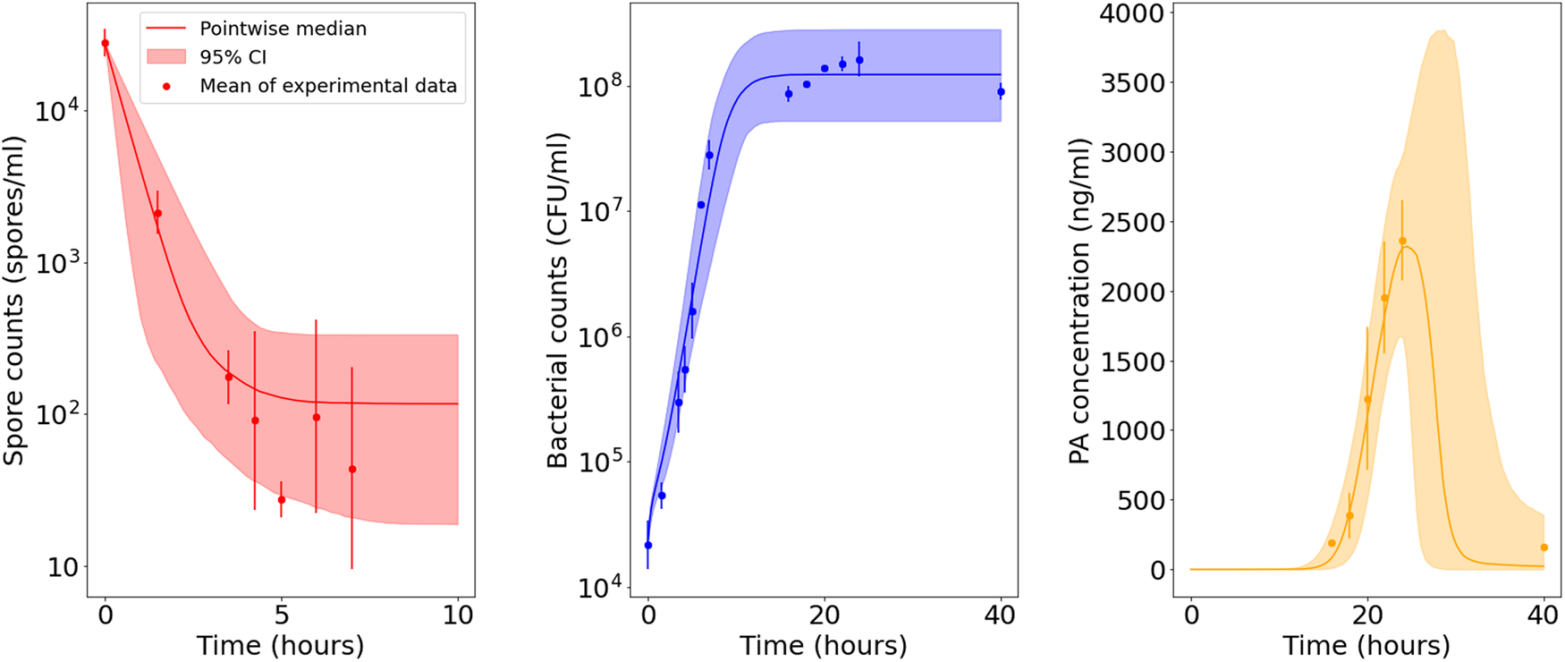
Model posterior predictions corresponding to the Dstl data set. Pointwise medians (solid lines) and 95% credible intervals (shaded regions) of the model posterior predictions are shown for *S*(*t*)+(1–*f*)S^*^_0_ (left), *B*(t) = *N*(*t*)+*V*(*t*) (middle), and *P*(*t*) (right), using the parameter posterior distribution in Fig. 10. The experimental data used to fit the model are presented as mean ± standard deviation from three independent experiment runs, obtained from the Dstl experiment described in the Methods section.

**Fig. 12:**
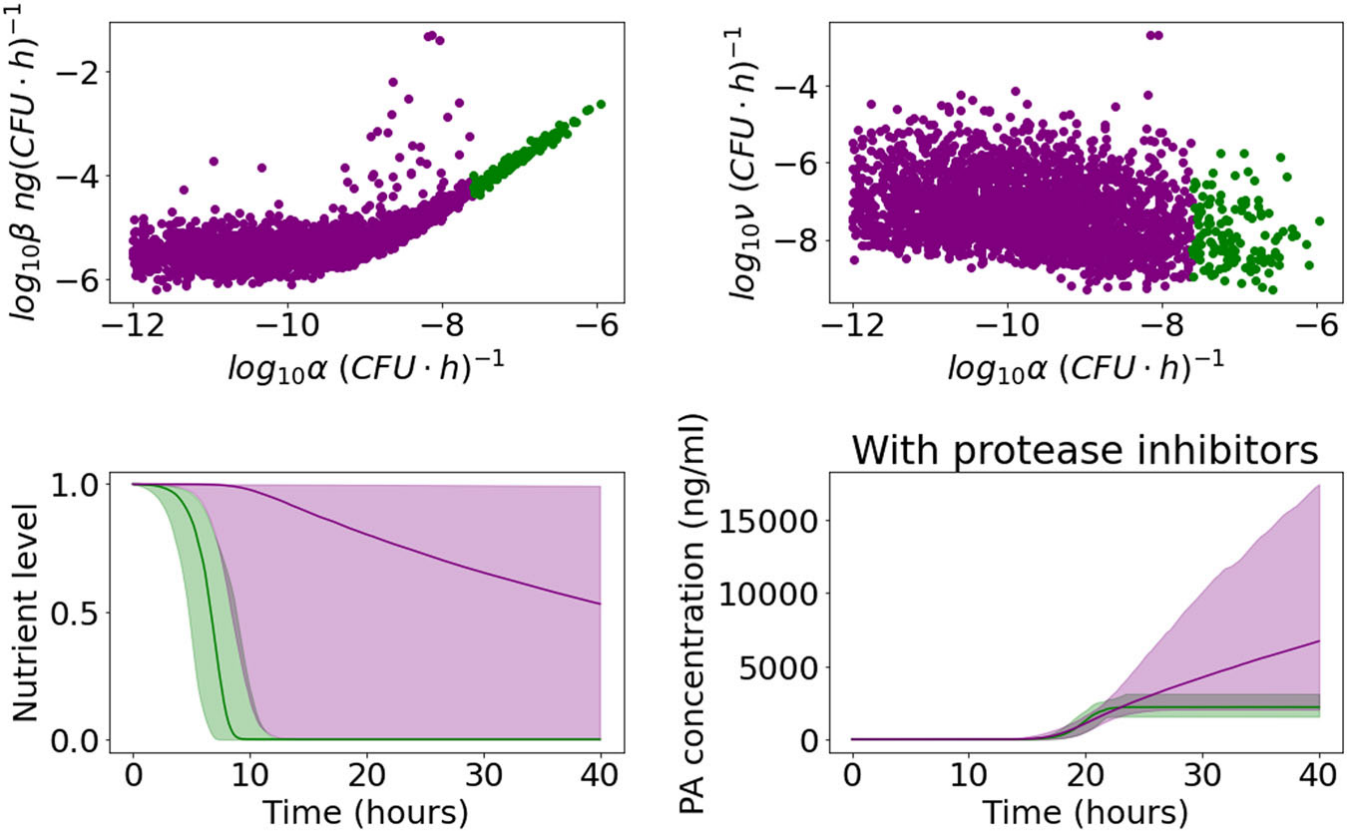
Distinguishing between parameter regimes corresponding to a fast or slow decline in nutrient level, for the Dstl posterior distribution. Top row: scatter plots of the parameter sets in the posterior sample from the calibration with the Dstl data set. The value of *α* is plotted on the *x* axis and on the *y* axis is *β* (left) or *ν* (right). Points are coloured green if the value of *α* is larger than the minimum value of *α* in the posterior sample from the calibration with Zai et al.^7^ A16R strain data set (${\log }_{10}\alpha =-7.6$), and are coloured purple if the value of *α* falls below this threshold. Bottom row: Pointwise medians (solid lines) and 95% credible intervals (shaded regions) of the model posterior predictions for *G*(*t*) (left) and *P*_*i*_(*t*) (right) using the posterior parameter sets with ${\log }_{10}\alpha \ge -7.6$ (green) or ${\log }_{10}\alpha < -7.6$ (purple).

The model predictions and data in Fig. 11 show quicker bacterial growth and a higher steady-state level of bacteria compared with the other experiments. The vegetative bacterial growth curve is determined by the parameters *λ* and *K*. While the estimates for these parameters are fairly consistent between the Zai et al.^7^ and Charlton et al.^10^ experiments, we estimate a faster bacterial growth rate, *λ*, and a higher carrying capacity, *K*, for the Dstl data set. One possible explanation is that the richer BHI medium used in the Dstl experiment may have enabled the bacteria to divide faster and grow to a higher concentration. Most of the other marginal posterior distributions shown in Fig. 10 are fairly consistent with those of the Zai et al.^7^ Sterne strain data set. Similar to what we observed from the calibration with the Zai et al.^7^ Sterne strain data set, Fig. 12 shows that we can again identify two parameter regions that show different correlations between *α* and *β* or *ν*. These two parameter regions lead to contrasting predictions for the nutrient level and the PA concentration in the presence of protease inhibitors. For parameter sets that have values of *α* consistent with the posterior sample from the Zai et al.^7^ A16R strain calibration, the model predicts that nutrients will become fully depleted and that the PA concentration will plateau in the absence of PA degradation by bacterial proteases (i.e., in the presence of protease inhibitors). On the other hand, accepted parameter sets with much smaller values of *α* correspond to continual production of PA, but a larger estimated degradation rate by bacterial proteases means that the model can still capture the observed decline in PA in the Dstl experiment.

We note that the delay in PA production by each bacterial CFU and the delay in the production of proteases by the bacteria, given by *τ*_1_ and *τ*_2_, respectively, are estimated to be slightly longer in our experiment. This may be because spores were directly inoculated into the assay culture at the start of the experiment, whereas Zai et al.^7^ used a bacterial culture that had already been growing for 24 hours. Furthermore, we did not heat activate the spores prior to inoculation, as was done by Charlton et al.^10^.

## Discussion

We propose a DDE model of the in vitro dynamics of *B. anthracis* growth and PA production and degradation. Making use of a new experimental data set obtained at Dstl during this study, as well as two other independent data sets by Zai et al.^7^ and Charlton et al.^10^, we have carried out parameter calibration for each data set by means of ABC-SMC^11^. We used a common set of mathematical equations to model each data set, which is flexible enough to describe the dynamics of different strains and culture conditions. We have then compared the estimated parameter values across data sets and explained possible reasons for the observed differences. Many of the parameters are consistently estimated across different data sets, but there are a few notable differences (see Fig. 2). For example, as discussed above, the data from the Dstl experiment shows quicker bacterial growth, reaching a higher steady-state level. We hypothesise that this could be due to differences in the culture medium used in the experiments. Furthermore, a much higher PA yield was obtained in the Charlton et al.^10^ experiment, which is reflected in the corresponding parameter estimates. This may be due to the method of static incubation implemented by Charlton et al.^10^. It is unclear why we observed strain differences between Sterne and A16R regarding growth rate and time to PA or protease production. However, one plausible explanation might be that Sterne’s rich history in laboratory studies and vaccine production has passaged it into being better suited for growth under these conditions. It is noteworthy, however, that the differences observed between strains within the study by Zai et al.^7^ are not more significant than the differences between the different experimental studies using the same strain. This indicates that, as predictors for growth and PA production, strain differences are only as influential as the many and small nuances associated with inter-laboratory practices.

An important feature of the mathematical model proposed here is that it distinguishes between natural PA decay and that caused by proteases secreted by the bacteria, such as InhA1. Decay due to proteases is implicitly included in the model via a term in which the rate of PA removal is assumed to be proportional to the number of vegetative bacteria that were present in the culture *τ*_2_ hours ago, where *τ*_2_ represents a delay taken for the bacteria to produce proteases. In the study by Zai et al.^7^ using the A16R strain, data from the experiment with protease inhibitors allowed us to more accurately estimate the PA decay rate in the absence of protease effects, given by *ν*_0_. We then leveraged the information gained about the natural decay rate of PA to set a value for *ν*_0_ when calibrating the model to the other data sets. However, when calibrating the model to data sets that did not include data from an experiment using protease inhibitors, we found that the observed decline in PA could be explained by two different parameter regimes, corresponding to a fast or slow decline in nutrient level. If the nutrients are consumed quickly, PA production eventually stops, and the PA concentration decreases due to PA degradation. On the other hand, if nutrients are consumed more slowly (and hence the PA production rate only decreases slightly), a much higher rate of PA degradation is needed to capture the decline in PA concentration. Additional experiments could help to choose among these different parameter regimes that have been identified, and the specific mechanisms that can explain the observed PA decay. However, our preliminary results seem to favour the depletion of nutrients as the main mechanism behind the rapid decay in PA concentration observed in most data sets at late times.

In our mathematical model, protease concentration is not explicitly modelled as a variable, since we are limited in the available experimental data. However, if future experiments were able to additionally obtain protease measurements, then the model could be adapted to include a more detailed description of the production of proteases, and their action on proteins produced by the bacteria (e.g. PA). In addition to in vitro experiments, future work should aim to discover whether these enzymes are also produced in vivo. The PA production rate predicted by the in vitro modelling results presented here may also be accurate in vivo. However, the degradation of PA is more uncertain within a host because there will likely be some degradation due to bacterial proteases, as well as proteases produced by the host.

The model variable that represents nutrient level is normalised by the initial nutrient level for each experiment, so that *G*(0) = 1. However, the type and amount of nutrients available to the bacteria will have varied significantly between the different studies. This implies that the estimated value of the maximal per CFU PA production rate, *β*, will depend on the nutrient availability in each individual experiment. In future, incorporating measurements of specific nutrients would help to unify the interpretation of parameters across studies. Furthermore, the model assumes that the per CFU PA production rate is proportional to the amount of nutrients. However, the relationship between nutrient level and PA expression by *B. anthracis* is likely to be more complicated than this. For instance, intermediate nutrient levels may provide the best environment for maximal PA production. Further experiments could be carried out with different specified nutrient levels, to quantitatively investigate the impact of nutrient level on PA expression. This would assist in the calibration of parameters that describe the relationship between nutrient level and PA expression and would also help to incorporate a more realistic description of this relationship into a mathematical model.

Production of the anthrax toxin proteins is a key factor in the within-host survival of *B. anthracis*. Lethal toxin and oedema toxin contribute to the severe symptoms suffered by a host infected by *B. anthracis*, since they impact numerous functions of the immune system, for example, by inhibiting the phagocytosis of bacteria by neutrophils. Quantifying PA production and degradation in vitro is an important step towards gaining a fuller understanding of in vivo toxin dynamics, since PA is the essential toxin component that facilitates binding of the other toxin proteins to cell surfaces. A benefit of the mechanistic modelling approach used here is that the underlying mechanisms of the model can be extended and modified as new scientific knowledge and data are generated. Furthermore, the mathematical model proposed here, and the parameter estimates obtained, could form a preliminary framework to be used in future within-host mathematical modelling efforts for anthrax. For instance, preliminary estimates obtained here for the bacterial growth rate and production rate of PA could be used to inform prior distributions when calibrating a within-host anthrax model with data from animal studies^16^. The understanding of PA dynamics gained through this study will also be valuable for the development of future mechanistic within-host models that incorporate medical treatments for anthrax, such as anti-toxin treatments. These types of models could be developed through coupling pharmacokinetic (PK) data that describes how the within-host concentration of the treatment will change through time^17,18^, with a pharmacodynamic (PD) description of the binding rate of PA as a function of anti-PA antibody concentration.

## Methods

### Dstl data set: growth of bacteria and viable counts

In this study, *B. anthracis* Sterne strain 34F2 from the Porton Down strain collection was used. Sterne (pXO1+, pXO2-) is un-encapsulated but retains the ability to produce toxins. 500 μl of Sterne spores, from a working stock containing 10^7^ CFU/ml, was inoculated into 50 ml Brain Heart Infusion (BHI) broth in a 250 ml Erlenmeyer flask to produce a culture containing 10^5^ CFU/ml. To this, sodium bicarbonate (Sigma-Aldrich) was added to a final concentration of 48 mM^19^. The culture was contained in a Biojar and a CO_2_ gas generator sachet (Scientific Laboratory Supplies) was added. The CO_2_ sachet was replaced after 24 hours of use as per the manufacturer instructions. Sodium bicarbonate and CO_2_ were added because both have been shown to increase the production of PA in vitro^19,20^ by helping to simulate the in vivo environment^21^. The culture was incubated at 37 °C with continuous shaking at ~182 rpm. Three independent growth experiments were performed in duplicate. In the first experiment, the culture was sampled at 0, 1.5, 3.5, 4.25, 5, 6, and 7 hours post-inoculation, in order to measure spore germination and bacterial growth. In the other two experiments, the culture was sampled at 16, 18, 20, 22, 24, 40, and 48 hours post-inoculation. At each time point, total viable counts were obtained by plating serial 10-fold dilutions (100 μl aliquots) onto L agar in triplicate. To obtain differential counts of the number of spores and vegetative bacteria in the culture, a sample was taken at each time point, diluted, and then heated to 70 °C for 30 minutes with vigorous shaking to kill vegetative cells^22^. The number of vegetative cells in the original sample was then calculated by subtracting the number of spores from the total CFU/ml. In the experiments where the culture was sampled at the 16, 18, 20, 22, 24, 40, and 48 hour time points, the culture was also filter sterilised at each time point by passing through a 0.22 μm syringe filter and stored at −20 °C before analysis using the automated western blot system, Jess^TM^ Simple Western (ProteinSimple, San Jose CA, USA).

### Dstl data set: automated western blot

Jess^TM^ Simple Western was used to quantify the production of PA during *B. anthracis* Sterne growth. Jess^TM^ automates the separation, probing, and detection of protein in a single hands-free assay. Recombinant PA (PharmAthene, Inc.) was diluted in 0.1 × Sample Buffer and Fluorescent 5 × master mix (ProteinSimple) to make samples with a final concentration ranging between 5 μg/ml–0.04 μg/ml to generate a standard curve. Filter sterilised supernatant from *B. anthracis* Sterne culture was mixed neat with Fluorescent 5 × master mix at a ratio of 4:1. All samples were then denatured by heating to 95 °C for 5 minutes. For all assays the 12–230 kDa Separation module (SM-W004, ProteinSimple) and Anti-Mouse Detection Module (DM-002, ProteinSimple) were used. Reagents were diluted and pipetted into the assay plate as per the manufacturer instructions. Jess^TM^ aspirates the reagents into glass capillaries before separating the HRP-conjugated MW ladder and sample by size. The sample proteins were immobilised to the capillary wall before immunoprobing with 1 μg/ml monoclonal PA4 primary antibody (2D4J, produced at Dstl) and HRP-conjugated anti-mouse secondary antibody. Luminol-Peroxide was added, and the chemiluminescent signal intensity from the PA target protein was represented graphically and as a virtual blot in the Compass Simple Western software (version 6.1.0, ProteinSimple). The signal intensity generated by PA was then interpolated against the standard curve to determine the concentration of PA at each time point of the Sterne growth experiment. Default assay conditions for a chemiluminescent 12–230 kDa size assay were chosen in the Compass Simple Western software, this included a sample separation time of 25 minutes and antibody incubation time of 30 minutes. Two negative system controls were run per assay, one containing no sample and the other containing no primary antibody, to check for cross-reactivity of reagents.

### Additional data sets

In addition to the experiments carried out at Dstl, described above, we have used data from two previously published studies, by Zai et al.^7^ and Charlton et al.^10^.

Zai et al.^7^ conducted similar experiments to the ones described above and measured bacterial growth and PA concentration for the A16R strain and the Sterne strain of *B. anthracis*, which are both un-encapsulated but retain the ability to produce the toxin proteins. Specifically, Erlenmeyer flasks each containing 100 ml of Luria-Bertani (LB) liquid medium were sterilised by autoclaving at 121 °C for 15 min and then warmed to 37 °C prior to inoculation with 1 ml of *B. anthracis* culture. The flasks were then incubated at 37 °C with vigorous agitation for up to 28 h. Culture supernatant samples were taken throughout the time course and used to obtain viable counts and quantify PA concentration. The viable counts of each strain are shown in ref. ^7^ [Fig. 1B] and the PA concentrations for each strain are shown in ref. ^7^ [Fig. 4A]. Zai et al.^7^ used a traditional western blot technique to qualitatively detect PA and then used an ELISA to quantify the concentration of PA, whereas we used a new automated western blot system (Jess^TM^) to quantify PA concentration.

Charlton et al.^10^ simulated the UK anthrax vaccine manufacturing process, which uses the Sterne 34F2 strain, and obtained in vitro data on bacterial growth and PA concentration. Specifically, Thompson bottles containing 450 ml of basal medium were sterilised by autoclaving at 121 °C for 15 min and then warmed to 37 °C prior to inoculation with 50 ml of a spore suspension that had a concentration of 2 × 10^4^ CFU/ml, giving an initial spore concentration in the Thompson bottles of 2 × 10^3^ CFU/ml. These Thompson bottles were then incubated statically at 37 °C for up to 32 hours. At various time points, three Thompson bottles were sacrificed, and the numbers of spore and bacterial CFU in the culture were measured. The PA concentration of the culture supernatants of each sacrificed bottle was determined using antigen-capture ELISA. Individual bottle sacrificing was used because repeated sampling from the same bottle was found to disturb the growing cultures. The viable counts are shown in ref. ^10^ [Fig. 1] and the PA concentrations are shown in ref. ^10^ [Fig. 4].

One of the main differences between our experimental methods and the ones used by Zai et al.^7^ is that they grew the *B. anthracis* bacteria for 24 hours before inoculating into the assay culture, whereas we did not grow the bacteria prior to inoculation and instead directly inoculated spores into the assay culture. Charlton et al.^10^ also directly inoculated spores into the assay culture, however, the spores had previously been heat-activated by heating the spore suspension at 60 °C for 60 minutes, whereas the spores we used had not been heat-activated. Another key difference between the experimental methods of the three studies is the type of culture medium used. Zai et al.^7^ inoculated bacteria into 100 ml of LB liquid medium, Charlton et al.^10^ used basal medium, and we used 50 ml of BHI broth and also added sodium bicarbonate and CO_2_. Finally, it may be important when interpreting the data to note that Charlton et al.^10^ incubated statically, whereas we and Zai et al.^7^ incubated with vigorous agitation.

### Mathematical model

We propose a deterministic, DDE model of the in vitro experiments described above, given by the following system of DDEs:

6 $$\begin{array}{ll}\frac{dS(t)}{dt}\;\;=-gS(t),\\ \frac{dN(t)}{dt}\;=gS(t)-mN(t),\\ \frac{dV(t)}{dt}\;=mN(t)+\lambda V(t)\left(1-\frac{V(t)}{K}\right),\\ \frac{dG(t)}{dt}\;=-\alpha V(t)G(t),\\ \frac{dP(t)}{dt}\;=\beta V(t-{\tau }_{1})G(t-{\tau }_{1})-({\nu }_{0}+\nu V(t-{\tau }_{2}))P(t).\end{array}$$

The model includes time-dependent variables to represent the number of germinating spores, *S*(*t*), newly desporulated bacteria, *N*(*t*), vegetative bacterial CFU, *V*(*t*), and the PA concentration (ng/ml), *P*(*t*), at time *t* ≥ 0. Due to the observation in all three in vitro data sets that the bacteria seem to down-regulate the production of PA at some point, and since it has been hypothesised to be due to a lack of resources such as glucose or amino acids in the culture medium^8,23^, we include an equation to represent the nutrient level, *G*(*t*), as a fraction of the initial level, with *G*(0) = 1.

*B. anthracis* spores cannot replicate, but first must undergo processes to convert into a vegetative cell^24^. These processes are collectively called germination, and they result in a delay before vegetative growth can occur. Once in a vegetative state, *B. anthracis* grows into chains of rod-shaped cells, with each chain measured as 1 CFU in the experiments. However, when a single spore first germinates, it takes time for the resulting newly desporulated bacterium to grow into a chain of cells. Thus, in the model, spores germinate (or desporulate) at rate *g* to become newly desporulated bacteria. The newly desporulated bacteria grow into vegetative bacterial chains with rate *m*. Then proliferation of the vegetative bacterial CFU is modelled by logistic growth, and these bacteria are assumed to use up nutrients in the culture medium with rate *α*, in order to produce PA. It is assumed that PA is produced by vegetative bacterial CFU, but it is not produced by the newly desporulated bacteria. The rate *β* can be interpreted as the maximal production rate of PA per bacterial CFU. When nutrients are in excess (i.e. *G*(*t*) ≈ 1), bacteria are assumed to produce PA at their maximal rate. As nutrients are used up by the bacteria, the per CFU production rate of PA reduces, proportional to the variable *G*(*t*).

Two terms contribute to the removal of PA. Firstly, the PA decreases at a rate proportional to the current amount of PA, where *ν*_0_ is the rate at which PA naturally decays. Secondly, it is believed that some of the PA will actively be digested due to proteases produced by the bacteria^7,9^. Therefore, a second mechanism of PA degradation is included in the model, in which the rate of PA removal is assumed to also be proportional to the number of vegetative bacterial CFU. Two time delays have been included in the PA equation. The delay *τ*_1_ represents the delay taken for bacteria to produce PA. The delay *τ*_2_ represents the delay taken for the bacteria to produce the proteases that degrade PA, where proteases are not explicitly modelled as a variable since no data is available for these.

The initial conditions of the model variables are given by,

$$S(0)=f{S}_{0}^{* },\,N(0)=\varepsilon {B}_{0}^{* },\,V(0)=(1-\varepsilon ){B}_{0}^{* },\,G(0)=1,\,P(0)=0,$$

where ${S}_{0}^{* }$ and ${B}_{0}^{* }$ are the number of spores and bacterial CFU measured (or inferred) at time zero, respectively. These depend on the experiment that is being modelled. It has been observed in these types of experiments that often, some small proportion of the initial spores never desporulate. Therefore, the parameter *f* represents the fraction of initial spores that are able to desporulate, which are the ones represented by variable *S*(*t*) in the model. The parameter *ε* represents the fraction of initial bacterial CFU that is in the newly desporulated state.

The only steady-state of the model is (*S*_*∞*_, *N*_*∞*_, *V*_*∞*_, *G*_*∞*_, *P*_*∞*_) = (0, 0, *K*, 0, 0). This state is stable, since all initial germinating spores and newly depopulated bacteria will eventually become vegetative bacteria, and the population of vegetative bacteria will approach the carrying capacity, *K*. Since the nutrients, *G*(*t*), are being used up by the vegetative bacteria, this variable will approach zero, at which point no more PA can be produced, and the PA will also decay to zero.

### Reporting summary

Further information on research design is available in the Nature Research Reporting Summary linked to this article.

### Supplementary information

Reporting summary

## Data Availability

The data generated in this study is provided in Table 3.
